# Texas Nature Observations

**Published:** 1917-01

**Authors:** 


### Book Reviews.

TEXAS NATURE OBSERVATIONS. By Dr. R. Menger, San Antonio. $2.00 per copy.

Dr. R. Menger, the author of this most valuable book on nature study, is a public benefactor, for the book has proven interesting to the laity, to doctors and lawyers, as well as to persons especially interested in nature study.

The doctor has done a splendid work in collecting so much valuable data concerning the fauna, fields, plants and streams of Texas. The book contains much valuable information which cannot be found in any other book.

The following complimentary notice appeared in the *American Review of Reviews:*

"One questions if we have not at least Fabres in the making when one comes upon such a rare and delightful book of nature knowledge as 'Texas Nature Observations and Reminiscences,' a work by Dr. R. Menger, San Antonio, Texas.

"This book is a vivid transcript of the author's impressions covering a space of many years of his personal observations of the living wild things of the Lone Star State and of her fields, plants and streams. * * * A more fascinating book for both old and young, or a better commentary on the excellent results both of knowledge and of mental and physical refreshment following the use of our leisure in the study of the out-of-door world can hardly be imagined."

---

Do you know that sunlight and sanitation, not silks and satins, make better babies?

